# Modelling of Electro-Viscoelastic Materials through Rate Equations

**DOI:** 10.3390/ma16103661

**Published:** 2023-05-11

**Authors:** Claudio Giorgi, Angelo Morro

**Affiliations:** 1 Department of Civil, Environmental, Architectural Engineering and Mathematics, University of Brescia, Via Valotti 9, 25133 Brescia, Italy; 2 Department of Informatics, Bioengineering, Robotics and Systems Engineering, University of Genoa, Via All’Opera Pia 13, 16145 Genova, Italy; angelo.morro@unige.it

**Keywords:** electro-viscoelastic materials, constitutive rate equations, thermodynamic consistency, electroelasticity with dielectric memory, ferroelectric hysteresis, 74F15, 74D10, 74A15, 74N30, 78-10, 80-10, 80A17

## Abstract

Models of dielectric solids subject to large deformations are established by following a thermodynamic approach. The models are quite general in that they account for viscoelastic properties and allow electric and thermal conduction. A preliminary analysis is devoted to the selection of fields for the polarization and the electric field; the appropriate fields are required to comply with the balance of angular momentum and to enjoy the Euclidean invariance. Next, the thermodynamic restrictions for the constitutive equations are investigated using a wide set of variables allowing for the joint properties of viscoelastic solids, electric and heat conductors, dielectrics with memory, and hysteretic ferroelectrics. Particular attention is devoted to models for soft ferroelectrics, such as BTS ceramics. The advantage of this approach is that a few constitutive parameters provide a good fit of material behaviour. A dependence on the gradient of the electric field is also considered. The generality and the accuracy of the models are improved by means of two features. The entropy production is regarded as a constitutive property per se, while the consequences of the thermodynamic inequalities are made explicit by means of representation formulae.

## 1. Introduction

Electro-viscoelastic dielectrics are a subject of recent research in connection with electrically sensitive materials, where an external electric field results in a large deformation. The coupling of electromagnetic fields in deformable materials has been developed within continuum mechanics; both the non-instantaneous response and the rate-dependent behaviour indicate that a viscoelastic scheme is in order. Various approaches to the mathematical modelling of electro-mechanical materials have been developed in the literature (see, e.g., [1,2] and refs therein). The following are some ideas applied for the modelling.

As is carried out in other domains of mechanics, in [3] the physical deformation gradient F is given a multiplicative decomposition into elastic $F_{e}$ and viscous $F_{v}$ parts; the Cauchy–Green tensor B and the stretching tensor D are decomposed accordingly. A restriction to electroelastic solids is developed in [4]; systematic use is made of the Maxwell stress while the electric field and the electric displacement are decomposed additively into applied and self fields. A nonlinear treatment of the interaction between electric field and deformation is established in [5] using systematically Lagrangian fields and letting the stress be decomposed into mechanical and ponderomotive parts while the ponderomotive part is the sum of polarization stress and Maxwell stress. An approach to electro-rheological materials is developed in [6] by accounting for dissipation through the stretching tensor D in a viscous-like scheme.

To establish a thermodynamically consistent scheme for dielectrics undergoing large strains, we follow some ideas that have lately been applied to hysteretic phenomena [7,8,9]. First, the entropy production is viewed as a constitutive property which has to be determined using a constitutive equation in accordance with the second-law inequality (see the systematic procedure developed in [10]). Further, consistent with the objectivity principle, the constitutive equations are required to be invariant under the group of Euclidean transformations. This leads to a selection of appropriate fields for the electric field and the polarization. Furthermore, representation formulae for vectors and tensors allow the maximal generality of the thermodynamic restrictions on the constitutive equations.

Owing to the deformation of the body, the representatives of the electric field and the polarization are found by requiring the Euclidean invariance and the validity of the balance of angular momentum; this, in fact, leads to a pair of Lagrangian vectors. Next, the thermodynamic consistency is developed in a way that allows for rate-type equations in several models: hypo-electroelasticity, electro-viscoelasticity, heat conduction (and electric conduction) in dielectrics, electroelasticity with dielectric memory, and ferroelectric hysteresis.

The aim of this paper is to show that the thermodynamic consistency, in addition to ascertaining the physical admissibility of the constitutive functions, is also a guideline to the setting of the material model, thus providing a simple scheme for the selection of the parameters characterizing the material behaviour. To validate the resulting models, it is necessary to compare the results with the experimental observations. The work is mainly devoted to theoretical aspects. Yet, in § 9.3, particular attention is paid to models for soft ferroelectrics, such as BTS ceramics (BaTiO${}_{3}$ doped with 7.5 mol% Sn). Virgin loops of Ba(Ti,Sn)O${}_{3}$ ceramics in dependence on the tin content are devised in ([11], Figure 6). Our model is able to very well describe materials of this type.

### Notation

Throughout, we denote by $\Omega \subset E^{3}$ the time-dependent region occupied by the body. The motion is described by the function χ on R×R with R the reference configuration and R the set of real numbers. Hence, Ω∋x=χ(X,t), X∈R, and we let $\nabla =\partial_{x},\nabla \;R\;=\partial_{X}$. The deformation gradient F is defined as F=∇Rχ or $F_{iK}=\partial_{X_{K}}\chi_{i}$. The function $v=\partial_{t}\chi$ provides the velocity. A superposed dot denotes the total time derivative; for any function f(x,t), we have $\dot{f}=\partial_{t}f+\left(v\cdot \nabla \right)f$. To avoid obvious ambiguities, the Green–Lagrange strain tensor is denoted by E (instead of E) and the stretching tensor by D (instead of D).

## 2. Balance Equations

We consider a ferroelectric, deformable body where dissipative properties are allowed to occur of a mechanical and electric character. To simplify the description of material properties, throughout, the electromagnetic fields are considered at the frame locally at rest with the body.

Let ρ and $\rho_{R}$ be the mass densities in Ω and R, respectively; the balance of mass leads to

$$\rho_{R}=J\rho .$$

Let T be the mechanical Cauchy stress tensor and b the mechanical body force. The equation of motion can be written in the form

$$\rho \dot{v}=\nabla \cdot T+\rho b+f_{P},$$

where $f_{P}$ is the force per unit volume of an electric character. In stationary conditions or in the approximation of a negligible magnetic field, we have $f_{P}=\left(P\cdot \nabla \right)E$, where E is the electric field and P the polarization (per unit volume). The balance of angular momentum results in

(1) skw(T+E⊗P)=0,

where $\mathrm{skw}A=\frac{1}{2}\left(A-A^{T}\right)$ denotes the skew part of a tensor A and ⊗ represents the dyadic product of two vectors, ${\left(E⊗P\right)}_{ij}=E_{i}P_{j}$. Let ε be the internal energy (per unit mass), L the velocity gradient, $L_{ij}=\partial_{x_{j}}v_{i}$, and p=P/ρ. Moreover, let q be the heat flux vector, *r* the energy supply (per unit mass), and J the electric current density. The balance of energy is expressed as

(2) $$\rho \dot{\varepsilon }=E\cdot J+\rho E\cdot \dot{p}+T\cdot L-\nabla \cdot q+\rho r.$$

Let θ be the absolute temperature and η the entropy density. The statement of the second law is: the inequality

(3) $$\rho \dot{\eta }+\nabla \cdot \frac{q}{\theta }-\frac{\rho r}{\theta }=\rho \gamma \geq 0$$

holds for any process compatible with the balance equations. The entropy production γ is assumed to be given by a constitutive function. Consequently, the process consists of η,q,r,γ and the other functions occurring in the balance equations.

Using the Helmholtz free energy

ψ=ε−θη

we can write the Clausius–Duhem inequality (3) in the form

(4) $$-\rho \left(\dot{\psi }+\eta \dot{\theta }\right)+J\cdot E+\rho E\cdot \dot{p}+T\cdot L-\frac{1}{\theta }q\cdot \nabla \theta =\theta \rho \gamma \geq 0.$$

### Representation Formulae

Let N be a given second-order tensor, |N|=1. Then, for any second-order tensor Z, we can write

$$Z=\left(Z\cdot N\right)N+Z_{⊥},\;Z_{⊥}\cdot N=0.$$

If $Z_{⊥}$ is unknown, then we can represent $Z_{⊥}$ in the form

$$Z_{⊥}=\left(I-N⊗N\right)G,$$

where I is the fourth-order unit tensor and G is an arbitrary second-order tensor. Once g=Z·N is given, we can write the representation formula

(5) Z=gN+(I−N⊗N)G.

Likewise, if z is a vector and we know the inner product z·n=f with a unit vector n, then we can represent z in the form

(6) z=fn+(1−n⊗n)w,

where w is an arbitrary vector.

## 3. Euclidean Invariance and Objectivity

A change in frame $F\to F^{*}$, given by a Euclidean transformation, maps $x\mapsto x^{*}$ in the form

(7) $$x^{*}=c+Qx,\;Q^{T}Q=1.$$

Under the transformation (7), F and E change as vectors,

$$F^{*}=QF,\;E^{*}=QE.$$

As shown in ([10], Ch.15), invariant scalars, vectors, and tensors may involve F and E. The right Cauchy–Green tensor C and the Green–Lagrange strain tensor E,

$$C=F^{T}F,\;E=\frac{1}{2}\left(C-1\right),$$

are invariant [12]. Instead,

$$L^{*}=QLQ^{T}+\dot{Q}Q^{T}$$

and then L is non-objective, namely, it is not a tensor relative to Euclidean transformations. By the standard decomposition

L=D+W,

where D is the stretching tensor and W is the spin, we have

$$D^{*}=QDQ^{T},\;W^{*}=QWQ^{T}+\dot{Q}Q^{T}.$$

Vectors and tensors under Euclidean transformations are said to be objective; D is an objective tensor, W is not objective.

Let

$$T_{RR}=JF^{-1}TF^{-T}$$

be the second Piola stress. Since $\dot{E}=F^{T}DF$ then

$$T\cdot D=J^{-1}\left(FT_{RR}F^{T}\right)\cdot D=J^{-1}T_{RR}\cdot \left(F^{T}DF\right)=J^{-1}T_{RR}\cdot \dot{E}.$$

Hence, we have

(8) $$T\cdot L=J^{-1}T_{RR}\cdot \dot{E}+T\cdot W.$$

The referential heat flux and temperature gradient

$$q_{R}=JF^{-1}q,\;\nabla \;R\;\theta =F^{T}\nabla \theta$$

are invariant and so is the power

(9) $$q\cdot \nabla \theta =J^{-1}q_{R}\cdot \nabla \;R\;\theta .$$

In connection with the electric field E and the polarization P, we can consider the fields

$$E=F^{T}E,\;\;P=JF^{-1}P;$$

E and P are the (Lagrangian) fields in reference configuration [13,14]. The fields E and P are invariant,

$$E^{*}={\left(F^{*}\right)}^{T}E^{*}=F^{T}Q^{T}QE=E,$$

$${\;P}^{*}=J^{*}{F^{*}}^{-1}P^{*}=JFQ^{T}QP=\;P;$$

since *J* is invariant, so are the fields $J^{p}E$ and $J^{q}\;P$ for any p,q∈Z. Moreover, ∇RE is an invariant while the scalar ${\left|\nabla E\right|}^{2}$ is an invariant scalar. The time derivative of invariants is invariant too and, hence, $\dot{E},\dot{\;P},{\dot{T}}_{RR},{\dot{q}}_{R}$ are invariants.

For later purposes, we represent the power $\rho E\cdot \dot{p}$ in terms of E and P. Since $\rho_{R}=\rho J$ and $P=J^{-1}F\;P$ then

$$p=\frac{1}{\rho }P=\frac{1}{\rho_{R}}F\;P,$$

whence

$$\dot{p}=\frac{1}{\rho_{R}}\left(\dot{F}\;P+F\dot{\;P}\right)=\frac{1}{\rho_{R}}\left(LF\;P+F\dot{\;P}\right)=\frac{1}{\rho }LP+\frac{1}{\rho_{R}}F\dot{\;P}.$$

It then follows that

$$\rho E\cdot \dot{p}=\left(E⊗P\right)\cdot L+J^{-1}E\cdot F\dot{\;P}.$$

Hence, we obtain

(10) $$\rho E\cdot \dot{p}=\left(F^{-1}E⊗F^{-1}P\right)\cdot \dot{E}+\left(E⊗P\right)\cdot W+J^{-1}E\cdot \dot{\;P}.$$

Incidentally,

(11) $$F^{-1}E⊗F^{-1}P=F^{-1}F^{-T}E⊗F^{-1}P=J^{-1}C^{-1}E⊗\;P.$$

Furthermore, by (10) and (11),

(12) $$\rho_{R}E\cdot \dot{p}=\left(C^{-1}E⊗\;P\right)\cdot \dot{E}+J\left(E⊗P\right)\cdot W+E\cdot \dot{\;P},$$

and

$$JT\cdot L=T_{RR}\cdot \dot{E}+JT\cdot W.$$

## 4. Constraint on the Stress Tensor

The fields E and P enjoy Euclidean invariance. We now examine the validity of the constraint (1) which accounts for the balance of angular momentum. Consider the Clausius–Duhem inequality in the form (4) and observe

$$-\dot{\psi }+E\cdot \dot{p}=\left(-\psi +E\cdot p\right)\dot{}-p\cdot \dot{E}.$$

Hence, we let

ϕ=ψ−E·p

and write inequality (4) in the form

$$-\rho \left(\dot{\phi }+\eta \dot{\theta }\right)-P\cdot \dot{E}+T\cdot L-\frac{1}{\theta }q\cdot \nabla \theta =\rho \theta \gamma \geq 0.$$

Let

θ,F,E,∇θ

be the set of variables for the functions ϕ,η,T,q,γ; of course, ϕ,η, and γ depend on F,E, and ∇θ through appropriate invariants. Substitution of $\dot{\phi }$ yields

$$-\rho \left(\partial_{\theta }\phi +\eta \right)\dot{\theta }+\left(T-\rho \partial_{F}\phi F^{T}\right)\cdot L-\left(P+\rho \partial_{E}\phi \right)\cdot \dot{E}-\rho \partial_{\nabla \theta }\phi \cdot \dot{\bar{\nabla \theta }}-\frac{1}{\theta }q\cdot \nabla \theta =\rho \theta \gamma \geq 0.$$

Owing to the arbitrariness of $\dot{\bar{\nabla \theta }},\dot{\theta }$ and $L,\dot{E}$, it follows that

$$\partial_{\nabla \theta }\phi =0,\;\eta =-\partial_{\theta }\phi$$

and

$$T=\rho \partial_{F}\phi F^{T},\;P=-\rho \partial_{E}\phi .$$

The constraint (1) results in

(13) $$\mathrm{skw}\;\partial_{F}\phi F^{T}=\mathrm{skw}\;E⊗\partial_{E}\phi$$

and the requirement (13) holds if $\partial_{F}\phi$ is related to $\partial_{E}\phi$.

To determine admissible relations, consider any objective field $\tilde{E}$ of the form f(J)E. Hence, we let ϕ depend on F through $E=(F^{T}F-1)/2$ and jointly on F and E through $\tilde{E}$; ${\tilde{E}}_{K}=f\left(J\right)F_{iK}E_{i}$. If $\phi =\phi (E,\tilde{E})$, then

$$\partial_{F}\phi F^{T}=F\partial_{E}\phi F^{T}+\partial_{{\tilde{E}}_{P}}\phi \partial_{F}{\tilde{E}}_{P}\;F^{T}.$$

Since

$$\partial_{F_{iK}}J=JF_{iK}^{-1},\;\partial_{F_{iK}}{\tilde{E}}_{P}=f^{\prime }E_{P}JF_{iK}^{-1}+fE_{i}\delta_{KP}$$

and

$$\partial_{F_{iK}}E_{PQ}=\frac{1}{2}\left(F_{iQ}\delta_{PK}+F_{iP}\delta_{QK}\right),$$

then

(14) $${\left(\partial_{F}\phi F^{T}\right)}_{ij}=F_{iP}\partial_{E_{PQ}}\phi \;F_{jQ}+\partial_{{\tilde{E}}_{P}}\phi f^{\prime }E_{P}J\delta_{ij}+f\partial_{{\tilde{E}}_{P}}\phi E_{i}F_{jP},$$

(15) $${\left(E⊗\partial_{E}\phi \right)}_{ij}=f\partial_{{\tilde{E}}_{P}}\phi \;E_{i}F_{jP},$$

where $f^{\prime }=df/dJ$. Now,

$$F\partial_{E}\phi F^{T}+f^{\prime }JE\cdot \partial_{\tilde{E}}\phi \;1\in \mathrm{Sym}.$$

Hence, using (14) and (15), it follows that the requirement (13) holds if and only if

$$\mathrm{skw}\partial_{F}\phi F^{T}=\mathrm{skw}E⊗\partial_{E}\phi ,$$

whence

$$fF\partial_{\tilde{E}}\phi =\partial_{E}\phi .$$

This relation holds identically for any electric field

$$\tilde{E}=f\left(J\right)F^{T}E.$$

In light of expression (12) of the power, the pair P,E seems more suitable to describe the electric behaviour in deformable bodies. That is why we then proceed with the choice of E, i.e., f=1, for the referential electric field. Consequently, we have

$$J\cdot E=J\cdot \left(F^{-T}E\right)=\left(F^{-1}J\right)\cdot E.$$

We assume J is a vector and then we might consider $J=F^{-1}J$ as an invariant current density, $J^{*}={\left(F^{-1}J\right)}^{*}={\left(QF\right)}^{-1}QJ=F^{-1}J=J$. Instead, for technical convenience, we consider $J=JF^{-1}J$ and, hence,

(16) $$J\cdot E=J^{-1}J\cdot E.$$

Incidentally, J equals the referential flux, say, $J_{R}$, as for any vector field, such as $q_{R}=JF^{-1}q$.

## 5. Thermodynamic Analysis

Motivated by the Euclidean invariance, we now investigate the Clausius–Duhem inequality (4) in the Lagrangian description. Hence, we consider *J* times inequality (4) and use the representations (8), (9), (10), and (16) of the powers T·L, q·∇θ, $\rho E\cdot \dot{p}$, and J·E to obtain

(17) $$\begin{array}{c} -\rho_{R}\left(\dot{\psi }+\eta \dot{\theta }\right)+J\cdot E+E\cdot \dot{\;P}+\left(T_{RR}+C^{-1}E⊗\;P\right)\cdot \dot{E}+J\left(T+E⊗P\right)\cdot W\\ -\frac{1}{\theta }q_{R}\cdot \nabla \;R\;\theta =\rho_{R}\theta \gamma \geq 0. \end{array}$$

Hereafter, we use the referential fields $\eta_{R}=\rho_{R}\eta$, $\psi_{R}=\rho_{R}\psi$. For later developments, it is convenient to consider the free energy

$$\phi_{R}=\psi_{R}-E\cdot \;P.$$

Moreover, to save writing, we let

(18) $$T_{RR}:=T_{RR}+C^{-1}E⊗\;P.$$

Using (11) and the definition of $T_{RR}$, we have

$$T_{RR}=J\left\{F^{-1}TF^{-T}+\left(F^{-1}E\right)⊗\left(PF^{-T}\right)\right\}=JF^{-1}\left\{T+E⊗P\right\}F^{-T}.$$

Consequently

(19) $$T_{RR}\in \mathrm{Sym}\;⟺\;T+E⊗P\in \mathrm{Sym}.$$

Equation (17) is then written as

(20) $$-\left({\dot{\phi }}_{R}+\eta_{R}\dot{\theta }\right)+J\cdot E-\;P\cdot \dot{E}+T_{RR}\cdot \dot{E}+J\left(T+E⊗P\right)\cdot W-\frac{1}{\theta }q_{R}\cdot \nabla \;R\;\theta =\rho_{R}\theta \gamma \geq 0.$$

### Constitutive Assumptions and Thermodynamic Restrictions

Viscoelasticity is a scheme that accounts for a persistent rate of the response under a constant action. This may suggest that we allow for rate equations of T,J,P, and q. Accounting for rate properties is also consistent with the modelling of hysteresis (in ferroelectrics). Thus, we might take (θ,F,E,P, $\nabla \theta ,T,q,\dot{F},\dot{E})$, as the set of independent variables. However, the dependence on the derivatives can occur only in an objective way. In particular, the function $\phi_{R}$ can depend only on Euclidean invariants. Hence, we let

$$\phi_{R}=\phi_{R}\left(\theta ,E,T_{RR},E,\;P,J,q_{R},\nabla \;R\;\theta ,\dot{E},\dot{E}\right)$$

and the like for $\eta_{R}$ and γ. The viscoelastic character is realized by letting ${\dot{T}}_{RR}$, ${\dot{q}}_{R}$, $\dot{\;P}$, and J be given by constitutive functions of

$$\Gamma =(\theta ,E,T_{RR},E,\;P,J,q_{R},\nabla \;R\;\theta ,\dot{E},\dot{E}).$$

The time derivative of $\phi_{R}$ is computed and substituted in (20) to obtain

(21) $$\begin{array}{c} -\left(\partial_{\theta }\phi_{R}+\eta_{R}\right)\dot{\theta }+\left(T_{RR}-\partial_{E}\phi_{R}\right)\cdot \dot{E}-\partial_{T_{RR}}\phi_{R}\cdot {\dot{T}}_{RR}-\left(\;P+\partial_{E}\phi_{R}\right)\cdot \dot{E}-\partial_{\;P}\phi_{R}\cdot \dot{\;P}\\ -\partial_{J}\phi_{R}\cdot \dot{J}-\partial_{q_{R}}\phi_{R}\cdot {\dot{q}}_{R}-\partial_{\nabla \;R\;\theta }\phi_{R}\cdot \nabla \;R\;\dot{\theta }-\partial_{\dot{E}}\phi_{R}\cdot \ddot{E}-\partial_{\dot{E}}\phi_{R}\cdot \ddot{E}\\ +J\cdot E+J\left(T+E⊗P\right)\cdot W-\frac{1}{\theta }q_{R}\cdot \nabla \;R\;\theta =\rho_{R}\theta \gamma , \end{array}$$

where γ≥0. The linearity and arbitrariness of $\nabla \;R\;\dot{\theta },\ddot{E},\ddot{E},\dot{\theta },W$ implies that

$$\partial_{\nabla R\;\theta }\phi_{R}=0,\;\partial_{\dot{E}}\phi_{R}=0,\;\partial_{\dot{E}}\phi_{R}=0,$$

(22) $$\eta_{R}=-\partial_{\theta }\phi_{R},\;T+E⊗P\in \mathrm{Sym}.$$

The symmetry condition in (22) is just the balance relation (1) of angular momentum. Thus, it follows that $T_{RR}\in \mathrm{Sym}$. Hence, (21) simplifies to

(23) $$\begin{array}{c} \left(T_{RR}-\partial_{E}\phi_{R}\right)\cdot \dot{E}-\partial_{T_{RR}}\phi_{R}\cdot {\dot{T}}_{RR}-\left(\;P+\partial_{E}\phi_{R}\right)\cdot \dot{E}-\partial_{\;P}\phi_{R}\cdot \dot{\;P}\\ -\partial_{q_{R}}\phi_{R}\cdot {\dot{q}}_{R}-\frac{1}{\theta }q_{R}\cdot \nabla \;R\;\theta =\rho_{R}\theta \gamma . \end{array}$$

Notice that $\dot{E},\dot{E},$ and ∇Rθ can take independent values and, so far, ${\dot{T}}_{RR},\dot{\;P},$ and ${\dot{q}}_{R}$ are functions of the whole set of variables Γ. This allows the possibility of cross-coupling effects, which are usually negligible. We then consider models arising from independent entropy productions, namely,

(24) $$\left(T_{RR}-\partial_{E}\phi_{R}\right)\cdot \dot{E}-\partial_{T_{RR}}\phi_{R}\cdot {\dot{T}}_{RR}=\rho_{R}\theta \gamma_{T}\geq 0,$$

(25) $$-\left(\;P+\partial_{E}\phi_{R}\right)\cdot \dot{E}-\partial_{\;P}\phi_{R}\cdot \dot{\;P}=\rho_{R}\theta \gamma_{E}\geq 0,$$

(26) $$-\partial_{J}\phi_{R}\cdot \dot{J}+J\cdot E=\rho_{R}\theta \gamma_{J}\geq 0,$$

(27) $$-\partial_{q_{R}}\phi_{R}\cdot {\dot{q}}_{R}-\frac{1}{\theta }q_{R}\cdot \nabla \;R\;\theta =\rho_{R}\theta \gamma_{q}\geq 0,$$

the entropy productions $\gamma_{T},\gamma_{E},\gamma_{J},\gamma_{q}$ being non-negative while $\gamma =\gamma_{T}+\gamma_{E}+\gamma_{J}+\gamma_{q}$. It is worth remarking that the decomposition (24)–(27) of the entropy production does not hinder joint mechanical-electrical-thermal effects due to the dependence of the constitutive functions on Γ.

## 6. Electroelastic and Hypo-Electroelastic Models

As to (24), we start with the simple case $\partial_{T_{RR}}\phi_{R}=0$ and $\gamma_{T}=0$. Hence, we have

$$\phi_{R}=\phi_{R}\left(\theta ,E,E,\;P,J,q_{R}\right),\;T_{RR}=\partial_{E}\phi_{R},$$

whence

$$T_{RR}=\partial_{E}\phi_{R}-C^{-1}E⊗\;P,$$

and

$$T=\rho \partial_{F}\phi \;F^{-T}-E⊗P.$$

If, instead, $\gamma_{T}>0$ then we may take

$$T_{RR}-\partial_{E}\phi_{R}=\Xi \;\dot{E},$$

where Ξ is a fourth-order positive definite tensor. Hence,

$$T_{RR}=\partial_{E}\phi_{R}+C^{-1}E⊗\;P+\Xi \;\dot{E},\;\rho_{R}\theta \gamma_{T}=\dot{E}\cdot \Xi \dot{E},$$

thus showing a symmetric term $\partial_{E}\phi_{R}$, a non-symmetric dielectric term $C^{-1}E⊗\;P$, and a viscous term $\Xi \;\dot{E}$. Apparently, $\partial_{E}\phi_{R}$ is a purely elastic term if $\phi_{R}=\phi_{1}\left(\theta ,E\right)+\phi_{2}\left(\theta ,E,\;P,J,q_{R}\right)$. The special case $\partial_{E}\phi_{R}=EE$ yields

$$T_{RR}=EE-C^{-1}E⊗\;P+\Xi \dot{E},$$

a generalization of the Kelvin–Voigt viscoelastic model with E the fourth-order elasticity tensor.

A different scenario follows from (24) when $\partial_{T_{RR}}\phi_{R}\neq 0$. Letting $\gamma_{T}=0$ (no dissipation), we have

$$\left(T_{RR}-\partial_{E}\phi_{R}\right)\cdot \dot{E}-\partial_{T_{RR}}\phi_{R}\cdot {\dot{T}}_{RR}=0,$$

and, hence, we can determine the expression of ${\dot{T}}_{RR}$ using the representation Formula (5) with $N=\partial_{T_{RR}}\phi_{R}/\left|\partial_{T_{RR}}\phi_{R}\right|$,

(28) $${\dot{T}}_{RR}=\frac{\left(T_{RR}-\partial_{E}\phi_{R}\right)\cdot \dot{E}-\rho_{R}\theta \gamma_{T}}{|\partial_{T_{RR}}\phi_{R}{|}^{2}}\partial_{T_{RR}}\phi_{R}+\left(I-\frac{\partial_{T_{RR}}\phi_{R}⊗\partial_{T_{RR}}\phi_{R}}{|\partial_{T_{RR}}\phi_{R}{|}^{2}}\right)G$$

for any second-order tensor function G of Γ. Let H be any non-singular fourth-order tensor function of Γ, deprived of $\dot{E}$, and choose $G=H\dot{E}$. Then, we have

(29) $${\dot{T}}_{RR}=E\dot{E},\;E=H-\frac{1}{|\partial_{T_{RR}}\phi_{R}{|}^{2}}\partial_{T_{RR}}\phi_{R}⊗\left[\partial_{E}\phi_{R}-T_{RR}+H^{T}\partial_{T_{RR}}\phi_{R}\right],$$

where E denotes a family of (possibly non-symmetric) fourth-order stiffness (or elastic) tensor functions parameterized by H. Equation (29) ascribes to $T_{RR}$ a hypoelastic character possibly parameterized by E and P. The corresponding stress

(30) $$T_{RR}=-C^{-1}E⊗\;P+T_{RR}$$

then consists of the electroelastic dyadic product $-C^{-1}E⊗\;P$ and the hypo-elettroelastic stress obeying the rate-type Equation (29).

A special but significant class of hypo-electroelastic models is obtained by assuming that $\partial_{E}\phi_{R}=0$. Consequently, (29) simplifies to

(31) $$E=H+\frac{1}{|\partial_{T_{RR}}\phi_{R}{|}^{2}}\partial_{T_{RR}}\phi_{R}⊗\left[T_{RR}-H^{T}\partial_{T_{RR}}\phi_{R}\right].$$

We can look for H≠0 such that

(32) $$T_{RR}-H^{T}\partial_{T_{RR}}\phi_{R}=0$$

holds identically. If this is so, it follows from (31) that E=H, thus eliminating the dyadic term and then

(33) $${\dot{T}}_{RR}=H\left(\theta ,E,E,\;P,J,q_{R}\right)\dot{E}.$$

For definiteness, we now assume the free energy in a quadratic form with respect to $T_{RR}$. Let $G_{0}=G_{0}^{T}$ be a non-singular fourth-order tensor and set

$$\phi_{R}=\Phi_{R}\left(\theta ,E,\;P\right)+\frac{1}{2}T_{RR}\cdot G_{0}^{-1}\left(\theta ,E,\;P\right)T_{RR}.$$

Since $\partial_{T_{RR}}\phi_{R}=G_{0}^{-1}T_{RR}$, condition (32) reads

$$T_{RR}-H^{T}G_{0}^{-1}T_{RR}=0.$$

Due to the symmetry of $G_{0}$ and the arbitrariness of $T_{RR}$, we conclude that $H=G_{0}$ so that (33) becomes

$${\dot{T}}_{RR}=G_{0}\dot{E}.$$

If, further, we assume $G_{0}$ is a constant tensor, then an integration allows us to recover the linear electroelastic constitutive relation

$$T_{RR}=G_{0}E+T_{0},$$

where $T_{0}=T_{RR}{|}_{E=0}$ is an arbitrary initial value of the hypo-elettroelastic stress. Owing to (30), it follows

$$T_{RR}=G_{0}E+\left(1-C^{-1}\right)E⊗\;P+T_{RR}{|}_{E=0}.$$

## 7. Electro-Viscoelastic Models

If $\partial_{T_{RR}}\phi_{R}\neq 0$ and $\gamma_{T}>0$ then Formula (5) with $N=\partial_{T_{RR}}\phi_{R}/\left|\partial_{T_{RR}}\phi_{R}\right|$ yields

(34) $${\dot{T}}_{RR}=\frac{\left(T_{RR}-\partial_{E_{RR}}\phi_{R}\right)\cdot \dot{E}-\rho_{R}\theta \gamma_{T}}{|\partial_{T_{RR}}\phi_{R}{|}^{2}}\partial_{T_{RR}}\phi_{R}+\left(I-\frac{\partial_{T_{RR}}\phi_{R}⊗\partial_{T_{RR}}\phi_{R}}{|\partial_{T_{RR}}\phi_{R}{|}^{2}}\right)G,$$

for any second-order function G. We can also write ${\dot{T}}_{RR}$ in the form

(35) $${\dot{T}}_{RR}=\frac{\left(T_{RR}-\partial_{E}\phi_{R}\right)\cdot \dot{E}-\rho_{R}\theta \gamma_{T}-\partial_{T_{RR}}\phi_{R}\cdot G}{|\partial_{T_{RR}}\phi_{R}|}N+G.$$

The corresponding stress

$$T_{RR}=-C^{-1}E⊗\;P+T_{RR}$$

then consists of the electroelastic dyadic product $-C^{-1}E⊗\;P$ and the electro-mechanical symmetric stress obeying a rate-type equation in the form (35) for a given G. Several viscoelastic schemes follow from (34) depending on the choice of $\phi_{R}$ and $\gamma_{T}$.

Solids are characterized by a stress dependence such that, asymptotically, $T_{RR}=G_{\infty }E$ with $G_{\infty }$, a positive-definite fourth-order tensor. Define

$$\phi_{R}=\Phi_{R}\left(\theta ,E\right)+\frac{1}{2}E\cdot G_{\infty }E+\frac{1}{2}\left(T_{RR}-G_{\infty }E\right)\cdot A\left(T_{RR}-G_{\infty }E\right),$$

and

$$\rho_{R}\theta \gamma_{T}=\alpha \left(T_{RR}-G_{\infty }E\right)\cdot A\left(T_{RR}-G_{\infty }E\right),\;\alpha \left(\theta ,E\right):={\left[\beta \left(\theta \right)+E\cdot \Lambda E\right]}^{-1},$$

where β>0, while A and Λ are positive-definite fourth- and second-order tensors. Notice that

$$\partial_{T_{RR}}\phi_{R}=A\left(T_{RR}-G_{\infty }E\right),\;N=\frac{A(T_{RR}-G_{\infty }E)}{\left|A(T_{RR}-G_{\infty }E)\right|},\;\partial_{E}\phi_{R}=G_{\infty }E-G_{\infty }A\left(T_{RR}-G_{\infty }E\right)$$

and let

$$A:=\frac{\left[T_{RR}-G_{\infty }E+G_{\infty }A\left(T_{RR}-G_{\infty }E\right)\right]\cdot \dot{E}-\alpha \left(T_{RR}-G_{\infty }E\right)\cdot A\left(T_{RR}-G_{\infty }E\right)]}{\left|A(T_{RR}-G_{\infty }E)\right|}.$$

Hence, the representation Formula (35) yields

$$\begin{array}{c} {\dot{T}}_{RR}=AN+\left(I-N⊗N\right)G\\ =\left[N\cdot \left(A^{-1}+G_{\infty }\right)\dot{E}-\alpha \left(T_{RR}-G_{\infty }E\right)\cdot N\right]N+G-\left(N⊗N\right)G\\ =G+\left(N⊗N\right)[\left(A^{-1}+G_{\infty }\right)\dot{E}-\alpha \left(T_{RR}-G_{\infty }E\right)-G]. \end{array}$$

Consequently, upon choosing

$$G=\left[A^{-1}+G_{\infty }\right]\dot{E}-\alpha \left(T_{RR}-G_{\infty }E\right),$$

we find

(36) $${\dot{T}}_{RR}-G_{\infty }\dot{E}+\alpha \left(T_{RR}-G_{\infty }E\right)=A^{-1}\dot{E}.$$

Equation (36) shows that $T_{RR}-G_{\infty }E$ evolves with a relaxation time τ that is influenced by the temperature and the electric field,

$$\tau =\frac{1}{\alpha }=\beta +E\cdot \Lambda E.$$

At constant strain, $\dot{E}=0$, the solution $T_{RR}$ to (36) asymptotically is

$$T_{RR}=G_{\infty }E,$$

as expected for a solid model. Finally, we note that, upon letting $G_{0}=A^{-1}+G_{\infty }$, we can write Equation (36) in the form

$${\dot{T}}_{RR}+\frac{1}{\tau }T_{RR}=G_{0}\dot{E}+\frac{1}{\tau }G_{\infty }E,$$

thus obtaining the model of the standard linear solid.

## 8. Heat Conduction in Dielectrics

Equations (26) and (27) have the same structure and we then restrict attention to (27). We look at the evolution of $q_{R}$ and observe that the sought function ${\dot{q}}_{R}$ is subject to (27). This means that the thermodynamic restriction is confined to the inner product $\partial_{q_{R}}\phi_{R}\cdot {\dot{q}}_{R}$. Based on the representation Formula (6), we consider

$${\dot{q}}_{R}=\left({\dot{q}}_{R}\cdot n\right)n+\left(1-n⊗n\right)w.$$

Letting $\partial_{q_{R}}\phi_{R}\neq 0$ and choosing $n=\partial_{q_{R}}\phi_{R}/\left|\partial_{q_{R}}\phi_{R}\right|$ using (27), we have

(37) $${\dot{q}}_{R}=\left(\frac{1}{\theta }q_{R}\cdot \nabla \;R\;\theta +\rho_{R}\theta \gamma_{q}\right)\frac{\partial_{q_{R}}\phi_{R}}{|\partial_{q_{R}}\phi_{R}{|}^{2}}+\left(1-\frac{\partial_{q_{R}}\phi_{R}⊗\partial_{q_{R}}\phi_{R}}{|\partial_{q_{R}}\phi_{R}{|}^{2}}\right)w$$

Hence, the possible dependence of $\gamma_{q}$ and $\partial_{q_{R}}\phi_{R}$ on $T_{RR}$ and E allows the description of stress and electric field effects on heat conduction.

To show the flexibility of Equation (37) in the elaboration of (thermodynamically consistent) models, we show, e.g., how to obtain a Maxwell–Cattaneo equation. Let

$$\phi_{R}=\frac{1}{2}\lambda q_{R}^{2}+\cdots ,\;\rho_{R}\theta \gamma_{q}=\frac{1}{\theta \kappa }q_{R}^{2},\;w=\mu \nabla \;R\;\theta ,$$

where the dots denote quantities independent of $q_{R}$ while λ,κ,μ may depend on θ, E and $T_{RR}$. Since $\gamma_{q}\geq 0$, we let κ>0. Substitution into (37) yields

$${\dot{q}}_{R}=-\frac{1}{\lambda \theta \kappa }q_{R}-\frac{q_{R}\cdot \nabla \;R\;\theta }{\lambda \theta q_{R}^{2}}q_{R}+\mu \nabla \;R\;\theta -\mu \frac{q_{R}⊗q_{R}}{q_{R}^{2}}\nabla \;R\;\theta .$$

The two terms with $q_{R}\cdot \nabla \;R\;\theta$ cancel by letting μ=−1/λθ. With this value of μ we find the equation

$${\dot{q}}_{R}=-\frac{1}{\lambda \theta \kappa }q_{R}-\frac{1}{\lambda \theta }\nabla \;R\;\theta$$

which is in the Maxwell–Cattaneo form with relaxation time

τ=λθκ.

In stationary conditions we have the Fourier-like equation

$$q_{R}=-\kappa \nabla \;R\;\theta ;$$

the positive value of κ required by $\gamma_{q}\geq 0$ implies that the conductivity is positive.

## 9. Electroelastic Materials with Dielectric Memory

Equations (24) and (25) have the same mathematical structure. Hence, we can show that (25) allows hypo-electroelastic models as well as descriptions with memory for the past.

For formal simplicity we ignore the dependence on the electric current J and the heat flux $q_{R}$. Let $\phi_{R}:=\rho_{R}\phi \left(\theta ,E,T_{RR},E,\;P\right)$. Choosing $\gamma_{E}=0$, Equation (25) reduces to

$$\left(\partial_{E}\phi_{R}+\;P\right)\cdot \dot{E}+\partial_{\;P}\phi_{R}\cdot \dot{\;P}=0.$$

Assume $\partial_{\;P}\phi_{R}\neq 0$. Then, the representation Formula (6) can be applied by letting $n=\partial_{\;P}\phi_{R}/\left|\partial_{\;P}\phi_{R}\right|$ to obtain

$$\dot{\;P}=-\frac{\left(\;P+\partial_{E}\phi_{R}\right)\cdot \dot{E}}{|\partial_{\;P}\phi_{R}{|}^{2}}\partial_{\;P}\phi_{R}+\left(1-\frac{\partial_{\;P}\phi_{R}}{|\partial_{\;P}\phi_{R}|}⊗\frac{\partial_{\;P}\phi_{R}}{|\partial_{\;P}\phi_{R}|}\right)w.$$

We now restrict our attention to constitutive equations with linear dependence on $\dot{E}$. Hence, we let Ξ be any non-singular second-order tensor function of Γ, deprived of $\nabla_{R}\theta ,\dot{E}$, and $\dot{E}$, and consider $w=\Xi \dot{E}$. It follows that

(38) $$\dot{\;P}=\epsilon \left(\theta ,E,E,\;P\right)\dot{E},$$

where

$$\epsilon =\Xi -\frac{1}{|\partial_{\;P}\phi_{R}{|}^{2}}\partial_{\;P}\phi_{R}⊗\left[\;P+\partial_{E}\phi_{R}+\Xi^{T}\partial_{\;P}\phi_{R}\right].$$

Accordingly, ϵ can be viewed as a family of (possibly non-symmetric) permittivities in the form of second-order tensor-valued functions parameterized by Ξ. A particular model is obtained assuming that the Helmholtz free energy ψ is independent of P, and then $\partial_{\;P}\phi_{R}=-E$, so that

(39) $$\epsilon =\Xi +\frac{1}{{\left|E\right|}^{2}}E⊗\left[\;P+\partial_{E}\phi_{R}-\Xi^{T}E\right].$$

We can choose Ξ, and, hence, the function $\hat{\Xi }\left(\theta ,E,E,P,J,q_{R}\right)$, such that

(40) $$\;P+\partial_{E}\phi_{R}-{\hat{\Xi }}^{T}E=0$$

holds identically. Consequently, the dyadic term vanishes and Equation (38) simplifies to

$$\dot{\;P}=\hat{\Xi }\left(\theta ,C,E\right)\dot{E}.$$

Further, assume $\phi_{R}$ in the quadratic form

$$\phi_{R}=\Phi_{R}\left(\theta ,E,T_{RR}\right)+\frac{1}{2}E\cdot \Sigma \left(\theta ,C\right)E-E\cdot \;P,\;\Sigma =\Sigma^{T}.$$

Since $\partial_{E}\phi_{R}=\Sigma E-\;P$ then Equation (40) reads

$$\Sigma E-{\hat{\Xi }}^{T}E=0.$$

The validity of this relation for every vector E implies that $\hat{\Xi }=\Sigma$. The *paraelectric rate equation* follows by letting $\Sigma =\epsilon_{0}\chi_{R}$, where $\epsilon_{0}$ is the permittivity of free space and $\chi_{R}=JF^{-1}\chi F^{-T}$ is the electric susceptibility tensor in the material description. We then let

$$\dot{\;P}=\epsilon_{0}\chi_{R}\dot{E}.$$

As an example, consider a transversely isotropic material with easy axis m. The spatial electric susceptibility is then written in the form

$$\chi =\chi_{∥}m⊗m+\chi_{⊥}\left(1-m⊗m\right)=\chi_{⊥}1+\left(\chi_{∥}-\chi_{⊥}\right)m⊗m,$$

where $\chi_{∥}$ and $\chi_{⊥}$ are the electric susceptibilities in the direction parallel and perpendicular to m. Hence, it follows that

$$\chi_{R}=JF^{-1}\chi F^{-T}=\chi_{⊥}JC^{-1}+\left(\chi_{∥}-\chi_{⊥}\right)J^{-1}m_{R}⊗m_{R},\;m_{R}=JF^{-1}m.$$

If, instead, $\gamma_{E}>0$ then Equation (25) allows us to determine a large variety of dissipative electroelastic models. Assume $\partial_{\;P}\phi_{R}\neq 0$ and let $n=\partial_{\;P}\phi_{R}/\left|\partial_{\;P}\phi_{R}\right|$. The representation Formula (6) yields

(41) $$\dot{\;P}=-\frac{\left(\;P+\partial_{E}\phi_{R}\right)\cdot \dot{E}+\rho_{R}\theta \gamma_{E}}{|\partial_{\;P}\phi_{R}{|}^{2}}\partial_{\;P}\phi_{R}+\left(1-\frac{\partial_{\;P}\phi_{R}}{|\partial_{\;P}\phi_{R}|}⊗\frac{\partial_{\;P}\phi_{R}}{|\partial_{\;P}\phi_{R}|}\right)w.$$

Applying Equation (41), we now establish two relevant classes of models, dielectrics with memory and hysteretic dielectrics.

### 9.1. Dielectrics with Memory

They are characterized by a polarization dependence that shows relaxation and that, asymptotically, is given by $\;P=\epsilon_{0}\chi_{\infty }E$, with $\chi_{\infty }$, where $\chi_{\infty }$ is a positive-definite second-order tensor called *relaxation susceptibility*. To model this feature, we consider the free energy function

(42) $$\phi_{R}=\Phi_{R}\left(\theta ,E,T_{RR}\right)+\frac{1}{2}\epsilon_{0}E\cdot \chi_{R}E+\frac{1}{2}\left(\;P-\epsilon_{0}\chi_{R}E\right)\cdot A\left(\;P-\epsilon_{0}\chi_{R}E\right)-E\cdot \;P,$$

where A and $\chi_{R}$ are positive-definite second-order tensors while

$$\rho_{R}\theta \gamma_{E}=\alpha \left(\;P-\epsilon_{0}\chi_{\infty }E\right)\cdot A\left(\;P-\epsilon_{0}\chi_{\infty }E\right),\;\epsilon_{0}\chi_{\infty }=\epsilon_{0}\chi_{R}+A^{-1},$$

where α is a positive parameter possibly dependent on temperature and the scalar invariants of E and $T_{RR}$. Consequently,

$$\begin{array}{c} \partial_{\;P}\phi_{R}=A\left(\;P-\epsilon_{0}\chi_{R}E\right)-E=A\left(\;P-\epsilon_{0}\chi_{\infty }E\right),\\ \partial_{E}\phi_{R}=\epsilon_{0}\chi_{R}\left[E-A\left(\;P-\epsilon_{0}\chi_{R}E\right)\right]-\;P=-\epsilon_{0}\chi_{R}A\left(\;P-\epsilon_{0}\chi_{\infty }E\right)-\;P, \end{array}$$

and then

$$n=\frac{A(\;P-\epsilon_{0}\chi_{\infty }E)}{\left|A(\;P-\epsilon_{0}\chi_{\infty }E)\right|}.$$

For ease in writing, we define

$$B:=\frac{-\epsilon_{0}\left[\chi_{R}A\left(\;P-\epsilon_{0}\chi_{\infty }E\right)\right]\cdot \dot{E}+\alpha \left(\;P-\epsilon_{0}\chi_{\infty }E\right)\cdot A\left(\;P-\epsilon_{0}\chi_{\infty }E\right)}{\left|A(\;P-\epsilon_{0}\chi_{\infty }E)\right|}.$$

Hence, the representation Formula (41) can be written in the form

$$\dot{\;P}=-Bn+\left(1-n⊗n\right)w=w-\left(n⊗n\right)\left[-\epsilon_{0}\chi_{R}\dot{E}+\alpha \left(\;P-\epsilon_{0}\chi_{\infty }E\right)+w\right].$$

A simple model arises by letting the dyadic term vanish. This happens by choosing $w=\epsilon_{0}\chi_{R}\dot{E}-\alpha \left(\;P-\epsilon_{0}\chi_{\infty }E\right)$, in which case we find

(43) $$\dot{\;P}=\epsilon_{0}\chi_{R}\dot{E}-\alpha \left(\;P-\epsilon \chi_{\infty }E\right).$$

Since $\epsilon_{0}\chi_{R}=\epsilon_{0}\chi_{\infty }-A^{-1}$, then Equation (43) can be rewritten as

$$\dot{\;P}-\epsilon_{0}\chi_{\infty }\dot{E}+\alpha \left(\;P-\epsilon_{0}\chi_{\infty }E\right)=-A^{-1}\;\dot{E},$$

which shows a time rate of $\;P-\epsilon_{0}\chi_{\infty }E$ with relaxation time 1/α.

In essence, the models are characterized by the free energy ϕ and the entropy dissipation $\gamma_{E}$. It is of interest to show that, while maintaining the same free energy, we can model hysteretic phenomena by letting $\gamma_{E}$ be proportional to $|\dot{E}|$ (or $|\dot{\;P}|$).

### 9.2. Ferroelectric Hysteresis

Consider evolutions where only E and P are time-dependent while the remaining variables are constant. Restrict attention to cyclic processes in the time interval $\left[t_{i},t_{f}\right]$ with $\left(E,\;P\right)\left(t_{f}\right)=\left(E,\;P\right)\left(t_{i}\right)$, whence

$${\dot{\phi }}_{R}=\partial_{E}\phi_{R}\cdot \dot{E}+\partial_{\;P}\phi_{R}\cdot \dot{\;P},\;\phi_{R}\left(t_{f}\right)=\phi_{R}\left(t_{i}\right).$$

Integration in time of (25) yields

$$-\int_{t_{i}}^{t_{f}}\;P\cdot \dot{E}dt=\rho_{R}\theta \int_{t_{i}}^{t_{f}}\gamma_{E}\;dt\geq 0,$$

whence

(44) ∮P·dE≤0.

Hysteretic properties are now investigated by letting

$$\rho_{R}\theta \gamma_{E}=\zeta \left|\dot{E}\right|,\;\zeta >0.$$

Hence, P and E are subject to

(45) $$\left(\;P+\partial_{E}\phi_{R}\right)\cdot \dot{E}+\partial_{\;P}\phi_{R}\cdot \dot{\;P}=-\zeta \left|\dot{E}\right|.$$

Based on (41) and (45), we now determine a relation for $\dot{\;P}$. Let Λ be a second-order tensor. By selecting $w=\Lambda \dot{E}$, we have

$$\dot{\;P}=-\frac{n⊗(\;P+\partial_{E}\phi_{R})}{|\partial_{E}\phi_{R}|}\dot{E}-\frac{\zeta |\dot{E}|}{|\partial_{E}\phi_{R}|}n+\left(1-n⊗n\right]\Lambda \dot{E},$$

whence

(46) $$\dot{\;P}=\left[\Lambda -\frac{n⊗(\;P+\partial_{E}\phi_{R}+\Lambda^{T}\partial_{\;P}\phi_{R})}{|\partial_{\;P}\phi_{R}|}\right]\dot{E}-\frac{\zeta |\dot{E}|}{|\partial_{\;P}\phi_{R}|}n.$$

A simple particular case emerges by letting Λ such that

(47) $$\;P+\partial_{E}\phi_{R}+\Lambda^{T}\partial_{\;P}\phi_{R}=0,$$

thus implying the vanishing of the dyadic term. Equation (47) shows that a linear relation is required between $\partial_{E}\phi_{R}$ and $\partial_{\;P}\phi_{R}$.

In terms of the free energy (42), the requirement (47) reads

$$\left(\Lambda^{T}-\epsilon_{0}\chi_{R}\right)A\left(\;P-\epsilon_{0}\chi_{\infty }E\right)=0,$$

which implies $\Lambda =\epsilon_{0}\chi_{R}$. Hence, Equation (46) becomes

(48) $$\dot{\;P}=\epsilon_{0}\chi_{R}\dot{E}-\frac{\zeta |\dot{E}|}{|A\left(\;P-\epsilon_{0}\chi_{\infty }E\right){|}^{2}}A\left(\;P-\epsilon_{0}\chi_{\infty }E\right).$$

### 9.3. One-Dimensional Models of Hysteresis

Suppose that the body is transversely isotropic and the spatial fields E and P are collinear in the direction m of easy polarization. Let $\left(e_{1},e_{2},e_{3}\right)$ be an orthonormal basis where $e_{1}=m$ and assume $E=Ee_{1},P=Pe_{1}$. The deformation gradient is taken in the form

$$F=\mathrm{diag}\left(1+\nu ,1-\delta ,1-\delta \right),\;J=\left(1+\nu \right){\left(1-\delta \right)}^{2}.$$

Hence, we have

$$E=F^{T}E=\mathrm{diag}\left(\left(1+\nu \right)E,0,0\right),\;\;P=JF^{-1}P=\mathrm{diag}\left({\left(1-\delta \right)}^{2}P,0,0\right).$$

Consequently, E=(1+ν)E and $P={\left(1-\delta \right)}^{2}P$ are subject to

$$\dot{E}=\dot{\nu }E+\left(1+\nu \right)\dot{E},\;\dot{P}=2\left(1-\delta \right)\dot{\delta }P+{\left(1-\delta \right)}^{2}\dot{P}.$$

Owing to the transversely isotropic symmetry of the material, we let

$$\chi =\chi_{∥}e_{1}⊗e_{1}+\chi_{⊥}\left(1-e_{1}⊗e_{1}\right)$$

so that, in matrix form, $\chi =\mathrm{diag}(\chi_{∥},\chi_{⊥},\chi_{⊥})$. Hence, it follows that

$$\chi_{R}E=JF^{-1}\chi E={\left(1-\delta \right)}^{2}\chi_{∥}Ee_{1}.$$

Further, let

$$Ae_{1}=\alpha e_{1}.$$

We are then in a position to argue in a one-dimensional setting.

For small deformations (|ν|,|δ|≪1) and slow motions ($|\dot{\nu }E|\ll |\dot{E}|$, $|\dot{\delta }P|\ll |\dot{P}|$), we can follow the approximation E(t)≃E(t), P(t)≃P(t) for the time dependence. In rigid bodies E(t)=E(t), P(t)=P(t).

The one-dimensional version of (48) is

$$\dot{P}=\epsilon_{0}\chi_{∥}\dot{E}-\frac{\zeta }{\alpha [P-P(E)]}\left|\dot{E}\right|,$$

where $P\left(E\right)=\left(\epsilon_{0}\chi_{∥}+1/\alpha \right)E$. Except at inversion points (where $\dot{E}=0$), we can divide by $\dot{E}$ to obtain

(49) $$\frac{dP}{dE}=\epsilon_{0}\chi_{∥}-\frac{\zeta }{\alpha [P-P(E)]}\mathrm{sgn}\;\;\dot{E}.$$

If ζ is independent of $\dot{E}$ or depends on it at most through its sign, then (49) is invariant under the time change $t\to t^{*}=ct$, c>0. We can, therefore, say that the equation is rate-independent. By letting ζ=0, Equation (49) reduces to

$$\frac{dP}{dE}=\epsilon_{0}\chi_{∥}$$

where the right-hand side represents the differential permittivity of a paraelectric/dielectric material. In general, letting

(50) $$\epsilon_{1}=\epsilon_{0}\chi_{∥},\;\epsilon_{2}=-\frac{\zeta }{\alpha [P-P(E)]}$$

we can write Equation (49) as a differential equation,

(51) $$\frac{dP}{dE}=\epsilon_{1}+\epsilon_{2}\;\mathrm{sgn}\;\dot{E},$$

for the unknown function P(E). Hysteretic effects are described by the second term, $\epsilon_{2}\;\mathrm{sgn}\;\dot{E}$, in that the slope of P(E) changes depending on the sign of $\dot{E}$. Since $\epsilon_{2}$ is proportional to ζ, the vanishing of entropy production $\gamma_{E}$ implies $\epsilon_{2}=0$ and the corresponding model describes non-dissipative hypo-dielectric materials. Consequently, $\epsilon_{1}$, which possibly depends on the values of *P* and *E*, represents the slope of the paraelectric curve P(E). When $\epsilon_{2}\neq 0$, we can view (51) as the differential electric permittivity. Since it is usually observed to be non-negative, we then require that

$$\epsilon_{1}>0,\;\left|\epsilon_{2}\right|\leq \epsilon_{1}.$$

Since α,ζ>0, $\epsilon_{2}$ satisfies

(52) $$\epsilon_{2}\left\{\begin{array}{cc} >0 & \mathrm{if}\;P<P(E),\\ =0 & \mathrm{if}\;P=P(E),\\ <0 & \mathrm{if}\;P>P(E), \end{array}\right.$$

according to the counterclockwise sense required by ∮PdE≤0.

Summarizing the above analysis, we conclude that the model is characterized by three quantities: the paraelectric permittivity $\epsilon_{1}=\epsilon_{0}\chi_{∥}$, the hysteretic function ζ and the temperature-dependent function α. Since $\epsilon_{1}$ is fully determined by the free energy $\phi_{R}$ whereas $\epsilon_{2}$ depends also on ζ, different models can be obtained starting from the same function $\phi_{R}$. As we will see in a while, the function $\epsilon_{2}$, which is connected with the entropy production through ζ, governs the hysteretic properties of the material.

It is worth considering the case

$$\alpha \left(\theta \right)=\{\begin{array}{cc} \alpha_{0}/\left(\theta_{C}-\theta \right), & \mathrm{if}\;\theta \in (0,\theta_{C}),\\ 0, & \mathrm{otherwise}, \end{array}$$

where $\alpha_{0}>0$ depends on the characteristic parameters of the material, such as $\theta_{C}$ and $\chi_{∥}$. Since $P\left(E\right)=(\epsilon_{0}\chi_{∥}+\left[\theta_{C}-\theta \right]/\alpha_{0})E$ then

$$\lim_{\theta \to \theta_{C}}\epsilon_{2}=0,\;\lim_{\theta \to \theta_{C}}P\left(E\right)=\epsilon_{0}\chi_{∥}E.$$

We conclude that, regardless of the form of ζ, as $\theta \to \theta_{C}$ the curve P=P(E) represents the polarization curve of a paraelectric material.

### Ferroelectric Soft Materials

We present a simple example of the theory that appears appropriate for materials with “ferroelectrically soft" behaviour, such as BTS ceramics (BaTiO${}_{3}$ doped with a small amount of tin). They are distinguished by relatively high domain mobility and, thus, relatively easy polarization. The hysteresis loop of a soft material is, therefore, characterized by low coercive field strength and high spontaneous polarization.

Let P(E) be a monotone increasing odd function such that

$$\lim_{|E|\to +\infty }P^{\prime }\left(E\right)=1/\alpha .$$

Assuming $\zeta_{0}>0$ and

$$\zeta \left(E,P\right)=\zeta_{0}\epsilon_{0}\chi_{∥}\left(E\right){\left[P-P\left(E\right)\right]}^{2},\;\epsilon_{0}\chi_{∥}\left(E\right)=P^{\prime }\left(E\right)-1/\alpha ,$$

from (49) it follows that

$$\frac{dP}{dE}=\epsilon_{0}\chi_{∥}\left(E\right)\left[1-\tau_{h}\left(P-P\left(E\right)\right)\;\mathrm{sgn}\;\dot{E}\right],\;\tau_{h}=\frac{\zeta_{0}}{\alpha }>0.$$

The vanishing of $\chi_{∥}$ as |E| approaches infinity is a way of modelling the saturation property of hysteresis. Starting from different initial states $\left(E_{0},P_{0}\right)$, hysteresis cycles are obtained in the *E*-*P* plane by the system

$$\dot{P}=\left(P^{\prime }\left(E\right)-1/\alpha \right)[\dot{E}-\tau_{h}\left(P-P\left(E\right)\right)|\dot{E}\left|\right],\;\dot{E}=\omega A_{E}\cos\omega t.$$

**Figure 1:**
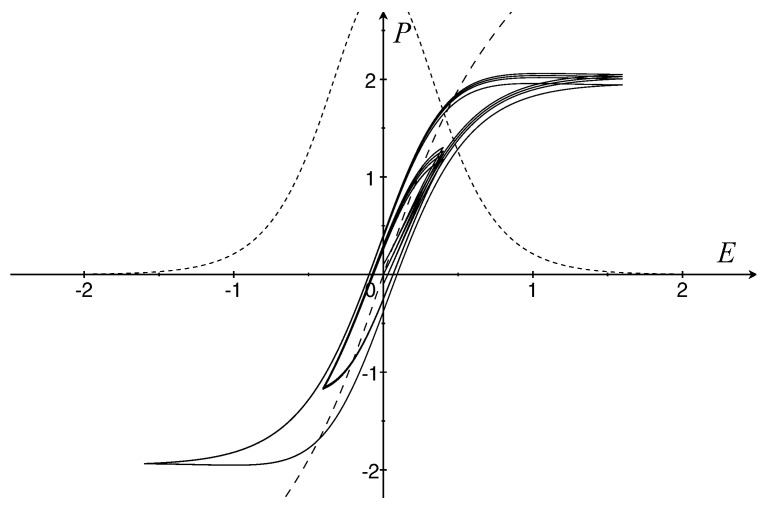
Graphs of the curves P (dashed) and $\chi_{∥}$ (short dashed); hysteresis loops (solid) starting from $\left(E_{0},P_{0}\right)$ where $E_{0}=0$ and $P_{0}=-0.1,\;0,\;0.1$.

Since the model considered here is rate-independent, the hysteresis loops are independent of the frequency ω at which the alternating electric field varies. In Figure 1, we choose

$$P\left(E\right)=\frac{1}{\alpha }\left[\tanh\left(2E\right)+E\right]$$

and $\alpha =\zeta_{0}=2/3$, so that $\tau_{h}=1$ and $\epsilon_{1}:=\epsilon_{0}\chi_{∥}\left(E\right)=3/\cosh^{2}\left(2E\right)$. Different amplitudes $A_{E}$ are used in order to highlight the shape of switching ($A_{E}=1.6$) and nonswitching ($A_{E}=0.4$) ferroelectric cycles.

Virgin loops of Ba(Ti,Sn)O${}_{3}$ ceramics in dependence on the tin content are devised in ([11], Figure 6). Their ferroelectric behavior changes from hysteretic (7.5 mol% Sn) to non-hysteretic (15 mol% Sn) polarization loops. Our model is able to very well describe materials of this type assuming that α is proportional to the molar content of tin. Indeed, as α increases the saturation limit is lowered and the hysteretic parameter $\tau_{h}$ tends to vanish. In particular, Figure 1 corresponds to Ba(Ti,Sn)O${}_{3}$ with 7.5 mol% Sn.

## 10. Dependence on the Gradient of the Electric Field

Spatial interaction in non-uniform electric fields are modelled by allowing for a dependence on the gradient of the electric (or the polarization) field [15,16]. For simplicity, we neglect heat conduction. We then let

$$\phi_{R}=\phi_{R}\left(\theta ,E,T_{RR},E,\;P,\dot{E},\dot{E},\nabla \;R\;E,\nabla \;R\;\nabla \;R\;E\right).$$

As we show in a moment, the dependence on ∇RE is allowed only if an extra-entropy flux occurs. The dependence on the second gradient ∇R∇RE is considered so that all of the constitutive functions depend a priori on the same set of variables.

The Clausius–Duhem inequality (21) is modified to

(53) $$\begin{array}{c} -\left(\partial_{\theta }\phi_{R}+\eta_{R}\right)\dot{\theta }+\left(T_{RR}-\partial_{E}\phi_{R}\right)\cdot \dot{E}-\partial_{T_{RR}}\phi_{R}\cdot {\dot{T}}_{RR}-\left(\;P+\partial_{E}\phi_{R}\right)\cdot \dot{E}-\partial_{\;P}\phi_{R}\cdot \dot{\;P}\\ -\partial_{\dot{E}}\phi_{R}\cdot \ddot{E}-\partial_{\dot{E}}\phi_{R}\cdot \ddot{E}-\partial_{\nabla \;R\;E}\phi_{R}\cdot \nabla \;R\;\dot{E}-\partial_{\nabla \;R\;\nabla \;R\;E}\phi_{R}\cdot \nabla \;R\;\nabla \;R\;\dot{E}\\ +J\left(T+E⊗P\right)\cdot W+\theta \nabla \;R\;\cdot k_{R}=\rho_{R}\theta \gamma , \end{array}$$

The arbitrariness of $\ddot{E},\ddot{E},\nabla \;R\;\nabla \;R\;\dot{E},\dot{\theta },W$ implies that

(54) $$\partial_{\dot{E}}\phi_{R}=0,\;\partial_{\dot{E}}\phi_{R}=0,\;\partial_{\nabla \;R\;\nabla \;R\;E}\phi_{R}=0,\;\eta_{R}=-\partial_{\theta }\phi_{R},\;T+E⊗P\in \mathrm{Sym}.$$

The possible dependence of $k_{R}$ on $\dot{E}$ allows us to write

$$\nabla \;R\;\cdot k_{R}=\partial_{\dot{E}}k_{R}\cdot \nabla \;R\;\dot{E}+...,$$

with the dots denoting terms independent of $\nabla \;R\;\dot{E}$. Hence, the arbitrariness of $\nabla \;R\;\dot{E}$ implies that

$$-\partial_{\nabla \;R\;E}\phi_{R}+\theta \partial_{\dot{E}}k_{R}=0.$$

Consequently,

(55) $$k_{R}=\frac{1}{\theta }\partial_{\nabla \;R\;E}\phi_{R}\dot{E}$$

to within terms independent of $\dot{E}$. For definiteness and simplicity, we let (55) hold exactly for $k_{R}$. In view of (55), we have

$$\nabla \;R\;\cdot k_{R}=\frac{1}{\theta }\partial_{\nabla \;R\;E}\phi_{R}\cdot \nabla \;R\;\dot{E}+\left[\nabla \;R\;\cdot \left(\frac{1}{\theta }\partial_{\nabla \;R\;E}\phi_{R}\right)\right]\cdot \dot{E}.$$

Hence, in light of (54), substitution of $\nabla \;R\;\cdot k_{R}$ in (53) yields

(56) $$\left(T_{RR}-\partial_{E}\phi_{R}\right)\cdot \dot{E}-\partial_{T_{RR}}\phi_{R}\cdot {\dot{T}}_{RR}-\left(\;P+\delta_{E}\phi_{R}\right)\cdot \dot{E}-\partial_{\;P}\phi_{R}\cdot \dot{\;P}=\rho_{R}\theta \gamma$$

where

$$\delta_{E}\phi_{R}=\partial_{E}\phi_{R}-\theta \nabla \;R\;\cdot \left(\frac{1}{\theta }\partial_{\nabla \;R\;E}\phi_{R}\right).$$

We notice that if, e.g., $\phi_{R}$ depends on ∇RE through ${\left|\nabla \;R\;E\right|}^{2}$ then $\partial_{\nabla \;R\;E}\phi_{R}\propto \nabla \;R\;E$ and, hence, $\delta_{E}\phi_{R}$ includes a term in $\Delta_{R}E$. That is why we started with a possible dependence on ∇R∇RE. As with (24)–(27), we notice that (56) holds if

$$\left(T_{RR}-\partial_{E}\phi_{R}\right)\cdot \dot{E}-\partial_{T_{RR}}\phi_{R}\cdot {\dot{T}}_{RR}=\rho_{R}\theta \gamma_{T},$$

(57) $$\left(\;P+\delta_{E}\phi_{R}\right)\cdot \dot{E}+\partial_{\;P}\phi_{R}\cdot \dot{\;P}=-\rho_{R}\theta \gamma_{E}.$$

The major interest in the dependence on ∇RE is given by (57) in view of the variational derivative $\delta_{E}\phi_{R}$. Hence, we apply the representation (6) to (57). Letting $n=\partial_{\;P}\phi_{R}/\left|\partial_{\;P}\phi_{R}\right|$, we have

$$\dot{\;P}=-\Lambda \dot{E}-\frac{\rho_{R}\theta \gamma_{P}}{|\partial_{\;P}\phi_{R}{|}^{2}}\partial_{\;P}\phi_{R}+\left(1-\frac{\partial_{\;P}\phi_{R}⊗\partial_{\;P}\phi_{R}}{|\partial_{\;P}\phi_{R}{|}^{2}}\right)w,$$

where

$$\Lambda =\frac{\partial_{\;P}\phi_{R}⊗\left(\;P+\delta_{E}\phi_{R}\right)}{|\partial_{\;P}\phi_{R}{|}^{2}}.$$

In the particular case of no dissipation, $\gamma_{E}=0$, and apart from the arbitrary contribution w, the representation shows a hypo-electric behavior,

$$\dot{\;P}=-\Lambda \dot{E}.$$

Here, though, the tensor Λ is also affected by ∇RE and ∇R∇RE.

## 11. Relation to Other Approaches

Hysteresis models of ferroelectric materials have been established through various approaches. Some models are based on hysteresis operators. This is, e.g., the case in [17] (see also [18,19]) where a Preisach operator P is considered in a one-dimensional setting, with a potential U dependent on an auxiliary state function q(ε,E), ε representing the strain and *E* the electric field. It is of interest that the assumed free energy involves a joint dependence on ε and *E* via U(E/f(ε)), which is quite analogous with the dependence of ζ on ${\left[P-P\left(E\right)\right]}^{2}$.

Thermodynamically consistent models are based on the compatibility with the second law of thermodynamics expressed by the Clausius–Duhem inequality. Yet the schemes adopted, and the mathematical procedure, are quite different to the present one in that they involve additional internal variables [20,21,22,23]. In [20], the thermodynamic potential, the enthalpy *H*, is a function of the strain ε, the electric field E, and an internal variable ξ. The strain ε and the electric displacement D are decomposed additively in reversible parts $\varepsilon^{e},D^{e}$ and remanent parts, $\varepsilon^{r},D^{r}$, with $D^{r}$ being equal to the polarization $P^{r}$. The Clausius–Duhem inequality is written in the form

$$\sigma \cdot \dot{\varepsilon }-D\cdot \dot{E}-\dot{H}\geq 0,$$

where σ is the Cauchy stress. Next, $\varepsilon ,E,\varepsilon^{r},P^{r}$ are taken as the variables and the inequality is splitted in

$$\sigma =\partial_{\varepsilon }H,\;D=-\partial_{E}H,\;\partial_{\varepsilon^{r}}H\cdot {\dot{\varepsilon }}^{r}+\partial_{P^{r}}H\cdot {\dot{P}}^{r}\leq 0,$$

as though $\varepsilon ,E,\varepsilon^{r},P^{r}$ were independent. The internal variable ξ is identified with the pair $\varepsilon^{r},P^{r}$ and, hence, the evolution of ξ is subject to

$$\tilde{\sigma }\cdot {\dot{\varepsilon }}^{r}+\tilde{E}\cdot {\dot{P}}^{r}\geq 0,$$

where $\tilde{\sigma }=-\partial_{\varepsilon^{r}}H$, $\tilde{E}=-\partial_{P^{r}}H$. By appealing to the principle of maximum remanent dissipation and introducing a non-positive switching function $\Phi (\tilde{\sigma },\tilde{E})$, it is found that

$${\dot{\varepsilon }}^{r}=\lambda \partial_{\tilde{\sigma }}\Phi \left(\tilde{\sigma },\tilde{E}\right),\;{\dot{P}}^{r}=\lambda \partial_{\tilde{E}}\Phi \left(\tilde{\sigma },\tilde{E}\right);$$

the loading/unloading conditions are $\lambda \geq 0,\Phi (\tilde{\sigma },\tilde{E})\leq 0$ and $\lambda \Phi (\tilde{\sigma },\tilde{E})=0$.

Ref. [21] models ferroelectric materials via the deformation gradient F, the electric field E, the temperature θ and an internal variable Q. In terms of the free energy Ψ it is found that

$$T_{R}=\rho_{R}\partial_{F}\Psi ,\;D=-\rho_{R}\partial_{E}\Psi ,\;\eta =-\partial_{\theta }\Psi$$

where $T_{R}$ is the first Piola stress, and

$$\beta \cdot \dot{Q}\geq 0,\;\beta :=-\rho_{R}\partial_{Q}\Psi .$$

The evolution of Q is then assumed to be governed by a dissipation potential function $\Phi (\dot{Q})$ so that

$$\beta =\partial_{\dot{Q}}\Phi$$

and the thermodynamic consistency is claimed to be achieved if Φ is a convex function.

The recourse to the function Φ, to characterize the evolution of the internal variable, may be viewed as the analogue of the present modelling directly via the entropy production γ. The advantage of our procedure is the consistent thermodynamic analysis via the Clausius–Duhem inequality. Furthermore, we avoid any decomposition of the pertinent fields, thus making the connection with experimental results more immediate.

## 12. Conclusions

This paper develops models of deformable dielectric solids. The models are quite general in that they account for viscoelastic properties and allow electric and thermal conduction. The procedure involves some conceptual points. First, the appropriate electric field and polarization are selected on the basis of two requirements: the fields are required to comply with the balance of angular momentum, skw(T+E⊗P)=0, and enjoy the Euclidean invariance. Among the possible choices we considered

$$E=F^{T}E,\;\;P=JF^{-1}P$$

as the invariant electric field and electric polarization.

Next, we investigated the thermodynamic requirements for constitutive equations involving

$$\theta ,E,T_{RR},E,\;P,J,q_{R},\nabla \;R\;\theta ,\dot{E},\dot{E},$$

where E is the Green–Lagrange strain, $T_{RR}=T_{RR}+C^{-1}E⊗\;P$, $C=F^{T}F$, and J is the referential current density. The resulting Equations (24)–(27) are examined using the representation formulae and this allows us to find the hypo-elastic and hypoelectric behaviour. Further, the entropy production γ is considered as a constitutive function, thus leading to models of hypo-electroelasticity, electro-viscoelasticity, heat conduction (and electric conduction) in dielectrics, electroelasticity with dielectric memory, and hysteretic dielectrics. As a sigificant generalization, often considered in the literature [16], we show the properties induced by considering the gradients ∇RE,∇R∇RE among the independent variables.

## Data Availability

Not applicable.
